# Draft genome sequence of an Acinetobacter albensis strain from untreated wastewater

**DOI:** 10.1099/acmi.0.001154.v5

**Published:** 2026-05-14

**Authors:** Kara D’Arcy, Alexander D. H. Kingdon, Anya Breen, Claudia McKeown, Ellie Allman, Priyanka Sharma, Amy McLeman, Adam P. Roberts

**Affiliations:** 1 Department of Tropical Disease Biology, Liverpool School of Tropical Medicine, Liverpool, UK

**Keywords:** *Acinetobacter*, cyanobactin, hydrocarbon degradation, mobile genetic elements, transposon, wastewater

## Abstract

We report the isolation and draft genome sequence of an *Acinetobacter albensis* strain C1-4G isolated from untreated wastewater from the Liverpool School of Tropical Medicine in Liverpool, UK. The 3.0 Mb genome contained several genes encoding alkane and aromatic hydrocarbon degrading enzymes, a *qacJ* antimicrobial resistance gene and several mobile genetic elements including a putative transposon, Tn*7860*, containing arsenic resistance genes. In addition, analysis of the biosynthetic gene clusters suggested the presence of genes that could enable the biosynthesis of baumannoferrin, a cyanobactin-type natural product, as well as a novel beta-lactone natural product.

## Data Summary

The data is available as part of BioProject PRJNA1161700. The raw reads have been deposited in SRA under the accession no. SRR31677847. The assembled and annotated genome has been deposited in GenBank under accession no. JBJXCW000000000.

## Introduction

*Acinetobacter* spp. are found ubiquitously in a range of natural environments, including soil and fresh water [1]. They have received a great deal of interest for their ability to biodegrade petroleum hydrocarbons, which could have potential for bioremediation of environmental pollution [2,4]. They are also of growing concern as a cause of opportunistic and nosocomial infections in humans, with a particular clinical focus on *Acinetobacter baumannii* [5,6]. *Acinetobacter albensis* was first identified from soil and water samples taken from protected landscape areas in the Czech Republic [7]. It has also been found in raw milk in Northwest Germany [8] and in Beijing and Heibei, China, [9] as well as in the Kayseri region of Turkey [10]. *A. albensis* has not been reported as a human pathogen [11]. There have only been two other *A. albensis* genomes uploaded to National Center for Biotechnology Information (NCBI), with a high ANI value of 97.5 but lower coverage of 78.27–80.94%, suggesting clear differences in the wider genomes. This draft genome adds to the information about the recently identified *A. albensis* species and presents the associated, potentially novel, transposon Tn*7860*, which encodes a putative arsenic resistance gene cluster.

## Isolation and assembly methods

*A. albensis* strain C1-4G was isolated from untreated wastewater collected from the outflow of the Liverpool School of Tropical Medicine (53° 25′ N 2° 58′ W, UK) [12] as part of the Swab and Send project [13]. Sterile swabs were used to spread wastewater samples onto brain heart infusion (BHI) agar, which were incubated at room temperature for 48 h. Individual colonies were then isolated from the plates and cultured statically in BHI broth for a further 48 h before being stored at −70 °C in BHI broth with 20% glycerol. 16S colony PCR using 16S PCR primers 27F (AGAGTTTGATCCTGGCTCAG) and 1492R (GGTTACCTTGTTACGACTT) was performed, followed by GENEWIZ (Azenta Life Sciences, https://www.genewiz.com/) performing Sanger sequencing. The closest 16S rRNA match (99.87% nucleotide identity, 100% coverage) was to *Acinetobacter* sp. TTH0-4 [14], a cold-active, crude oil degrading strain isolated from Qinghai–Tibet Plateau, China, NCBI accession no. CP012608. Restricting to the 16S RefSeq nucleotide sequences identified the closest match (99.8% nucleotide identity, 96% coverage) as *A. albensis* ANC 4874 (NR_145641.1) [15]. The difference in the isolation environments and the potential utility of hydrocarbon-degrading bacterial species led us to obtain the whole-genome sequence of this strain.

A single colony was cultured shaking at 160 r.p.m. in 12 ml BHI broth in a 50-ml Falcon tube at 28 °C until mid-log (around OD_600_ 1.0) was reached. An appropriate volume of cell culture was then centrifuged to give an OD_600_ equivalent to 10.0. The pellet was then resuspended in 500 µl DNA/RNA Shield (Zymo Research, USA) and sent to MicrobesNG (https://microbesng.com/) for short-read whole-genome sequencing. A portion of the cell suspension was lysed with Tris-EDTA buffer containing lysozyme, metapolyzyme and RNase A and incubated for 25 min at 37 °C. Proteinase K (0.1 ng ml^−1^) and SDS (0.5% v/v) were added and incubated for 5 min at 65 °C. The genomic DNA was then purified using solid-phase reversible immobilization beads and then resuspended in EB buffer (10 mM Tris-HCl, pH 8.0).

Library preparation was carried out using the Nextera XT Library Prep Kit (Illumina, USA) following the manufacturer’s protocol; however, the input DNA was increased twofold, and the PCR elongation time increased to 45 s. Short-read sequencing was then carried out on an Illumina NovaSeq 6000 (Illumina, USA) using a 250-bp paired-end protocol. The reads were adapter trimmed using Trimmomatic version 0.30 with a sliding window quality cutoff of Q15 [16]. This resulted in 540,863 raw reads. *De novo* assembly was performed using SPAdes version 3.7 [17], using QUAST version 5.2.0 to generate assembly quality metrics [18], and the contigs were annotated using NCBI’s Prokaryotic Annotation Pipeline version 6.8 [19]. Default settings were used throughout. CheckM was used to assess the genome completeness against the *Acinetobacter* CheckM marker set [20]. The average nucleotide identity (ANI) was calculated using the JSpeciesWS webserver [21]. The genome was analysed for biosynthetic gene clusters (BGCs) using antiSMASH version 7.0 bacterial version on the relaxed detection strictness setting [22]. The genome was analysed for antimicrobial resistance genes using ResFinder version 4.7.2 (>90% nucleotide identity, >60% coverage [23]) and CARD version 4.0.1 (strict matches only [24]), alongside using Mobile Element Finder version 1.0.3 [25]. Transposon designation was provided by the Transposon Registry [26].

## Genome description

*A. albensis* strain C1-4G’s genome had a total size of 3,042,601 bp and an overall G+C content of 38.5 mol% (Table 1). The mean coverage over the genome was 85.83×. Genome assembly resulted in 51 contigs with an N50 of 213,628. Across these contigs there were 2,910 predicted genes and 67 predicted tRNA sequences. The draft genome was compared to *A. albensis* ANC 4874 (FMBK00000000.1), resulting in an ANI of 98.02 at 87.50% coverage across the 3,018,180 bp genome. The *A. albensis* type strain had the highest ANI with *A. albensis* strain C1-4G, compared to the genomes of any of the other *Acinetobacter* spp. type strains; therefore, we have identified this as a strain of *A. albensis*. It was also compared to the closest 16S rRNA match, *Acinetobacter* sp. TTH0-4 (CP012608.1 [14]), to which it also had an ANI of 97.77 at 82.34% coverage. This difference may be due to adaptation to the different environments in which these three strains were isolated. *Acinetobacter* sp. TTH0-4, along with many *Acinetobacter* spp., contain a number of genes that could contribute to the degradation of alkanes and aromatic hydrocarbons [14,27, 28]. Five of which had high percentage nucleotide identities of 97% or higher in the described *A. albensis* strain C1-4G, including alkane 1-monooxygenase, 4-hydroxyphenylpyruvate dioxygenase, catechol 1,2-dioxygenases and long-chain-alcohol dehydrogenase 1.

**Table 1.** Description of the *A. albensis* C1-4G genome

Genome characteristic	*A. albensis* C1-4G
Number of raw reads	540,863
Draft genome size (bp)	3,042,601
Mean genome coverage	85.83×
G+C content (mol%)	38.5
Number of contigs	51
Number of contigs ≥1,000 bp	32
N50 (bp)	213,628
L50	6
Number of predicted genes	2,910
Number of predicted protein coding genes	2,778
Number of predicted tRNA sequences	67
Number of predicted rRNA sequences	3
Number of predicted pseudogenes	58
Completeness check (%)	99.21
Number of AMR genes (ResFinder, >90% identity, >60% coverage)	0
Number of AMR genes (CARD – strict matches only)	1
Number of mobile genetic elements (MGE, >90% identity, >95% coverage)	8
GenBank accession number	JBJXCW000000000
SRA accession number	SRR31677847

The genome was analysed for antimicrobial resistance genes using both CARD and ResFinder [23,24] and for mobile genetic elements (MGEs) using Mobile Element Finder [25]. For CARD, only one strict hit was identified, to the QacJ protein (ARO:3007014) with a 43% amino acid identity across 86% of the *A. albensis* C1-4G’s protein length, comprising 123 amino acids compared to 107 amino acids in the reference sequence. This suggests a small multidrug resistance efflux pump may be encoded by the genome, but as the identity was low, it may not function to confer antiseptic resistance [29]. However, this gene was also initially identified in Staphylococci, which could explain the low identity, and has more recently been widely found in *A. baumannii* [29,30]. For ResFinder, no matches were identified using the default search parameters.

**Fig. 1.**
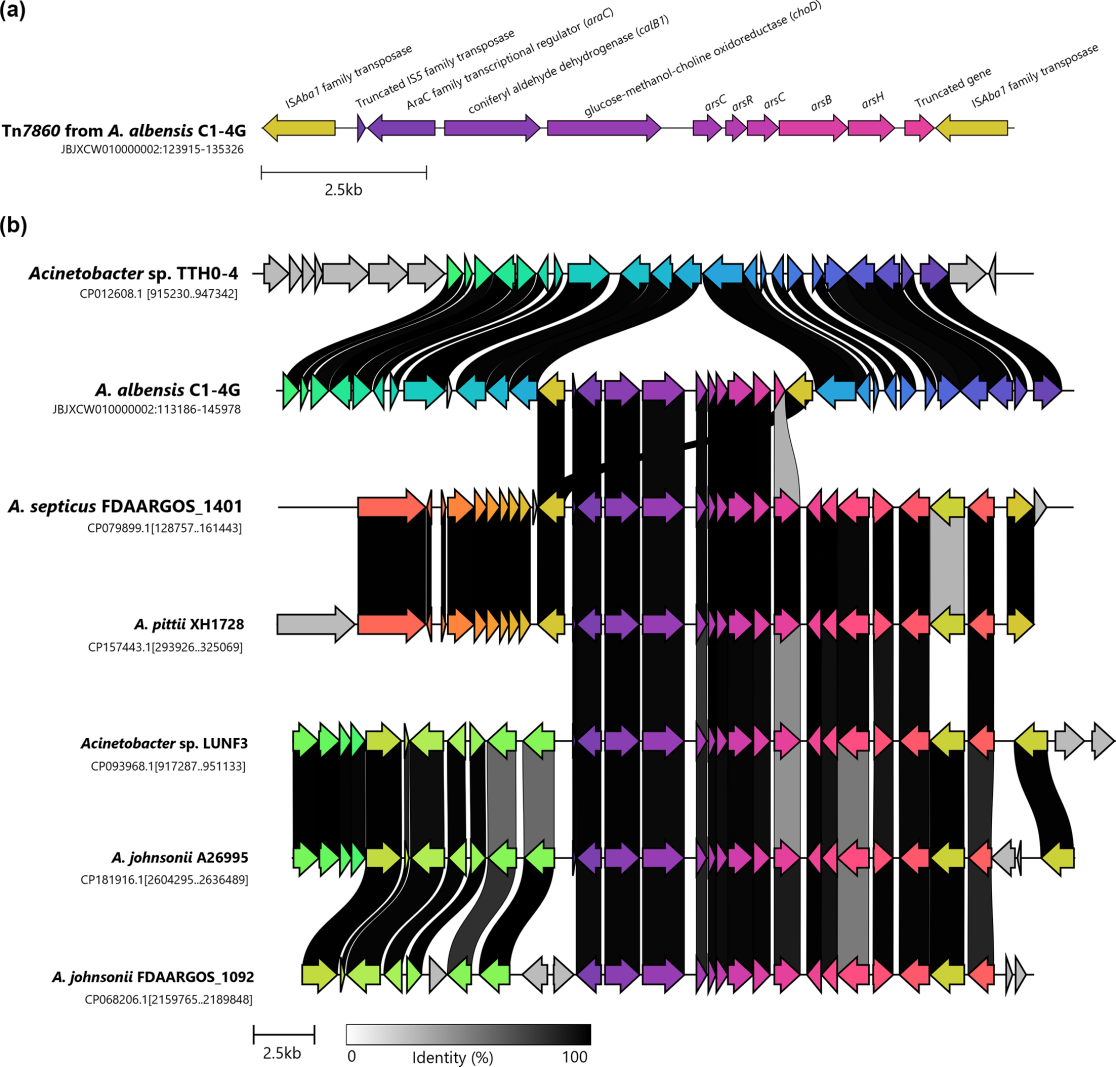
(**a**) Genes present on putative transposon Tn*7860*. (**b**) Comparison of the putative transposon Tn*7860*, between the *A. albensis* C1-4G strain reported herein and other *Acinetobacter* spp. Coloured genes indicate >40% nucleotide identity, and grey coloured genes indicate no close matches. Only the highest scoring match is used for colour and linkage. The figure was produced using clinker [45].

From Mobile Element Finder, eight MGEs were identified from four different families, IS*3*, IS*4*, IS*5* and IS*30*. These were specifically IS*Aba1*, IS*Aba21*, IS*Aba27*, IS*Aba31*, IS*Aba125* and IS*17*. There were two copies of IS*Aba1*, which had 100% nucleotide identity (100% coverage), and flanked a nine kb section of contig 2 (Fig. 1a). The putative transposon, Tn*7860*, contained several genes linked to arsenic resistance, including *arsB*, two *arsC*, *arsH* and *arsR* [31,32]. The remaining three genes on the transposon were annotated as encoding an AraC family transcriptional regulator, a coniferyl aldehyde dehydrogenase (*calB1*) and a glucose-methanol-choline oxidoreductase (*choD*). A truncated gene at the 3′ end of the transposon was predicted to encode a NAD(P)-binding domain. The genomic region which contained this putative transposon was compared with the equivalent region on the most closely related strain, *Acinetobacter* sp. TTH0-4 (Fig. 1b). This highlighted that the transposon was not present in this strain, while the genes directly surrounding the transposon in *A. albensis* C1-4G had between 97 and 100% nucleotide identity to the equivalent genes in *Acinetobacter* sp. TTH0-4. Searching the NCBI core nucleotide database using blastn [33], five matches with >95% nucleotide identity (76–90% coverage) were identified in other *Acinetobacter* spp. (Fig. 1b). These matches were against the genes present within the transposon, with only two hits including copies of the IS*Aba1* element. These two hits were part of a larger putative transposon, with one IS*Aba1* element adjacent to the same *araC* gene but the other one further downstream and including genes involved in amino acid degradation in the transposon. The top three closest matches (>97.5% nucleotide identity) were all present in opportunistic human pathogens, *Acinetobacter septicus*, *Acinetobacter pittii* and *Acinetobacter johnsonii* [11,34, 35]. All five hits contained copies of the IS*Aba1* MGE in other genomic locations.

**Fig. 2.**
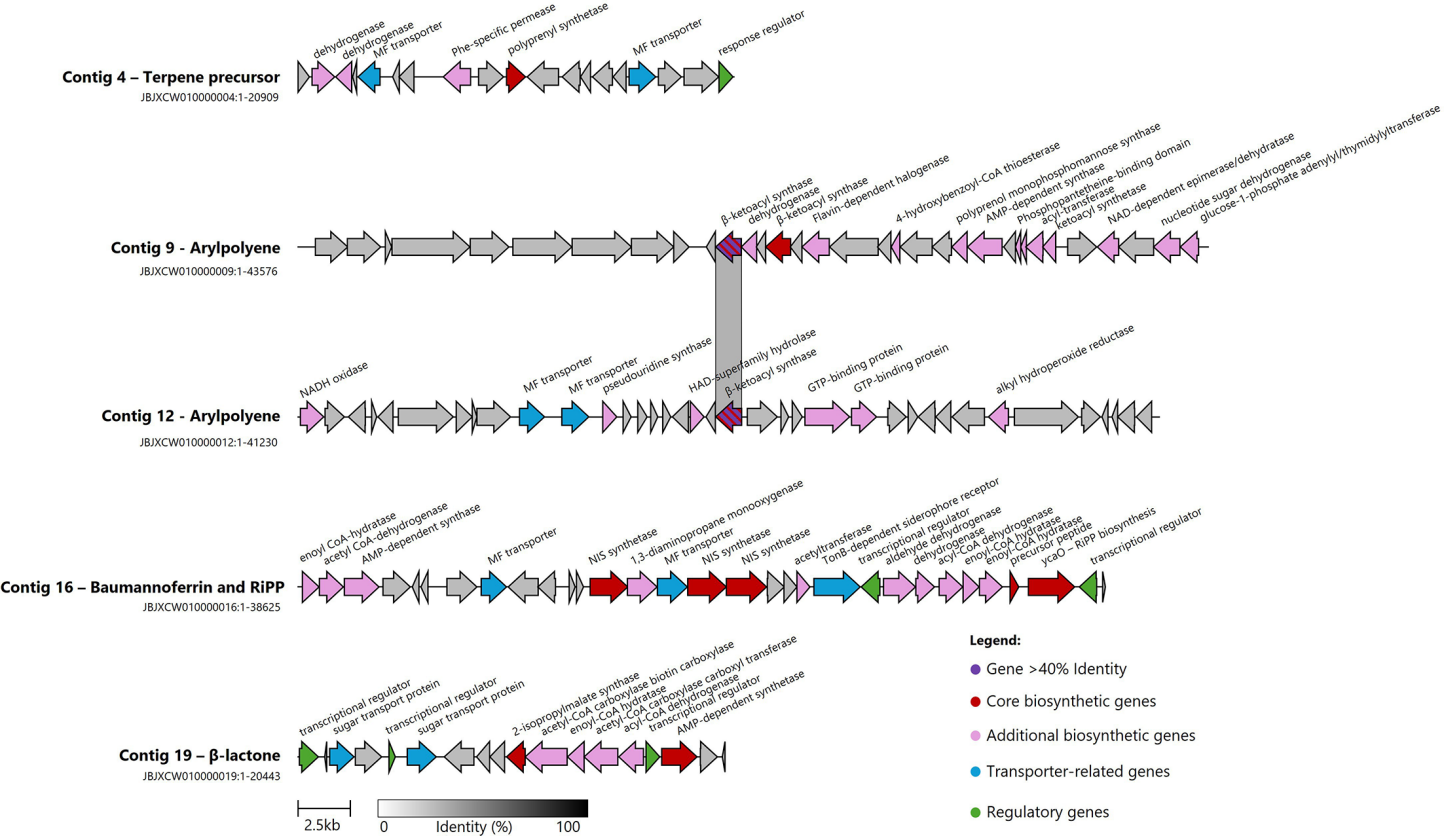
Comparison of the regions identified by AntiSMASH, which contained BGCs predicted to encode natural products, including baumannoferrin and an unknown (RiPP) cyanobactin-type compound. Gene functions were not included if the function was unknown but were included from antiSMASH based on unverified homology predictions. Coloured genes show antiSMASH grouping, with core biosynthetic genes as dark red, additional biosynthetic genes as lilac, transport-related genes as blue, regulatory genes as green and other genes in grey. The stripped pair of genes indicate >40% nucleotide identity. NIS, non-ribosomal peptide synthase independent siderophore; MF transporter, major-facilitator transporter. The figure was produced using clinker [45].

AntiSMASH was used to identify BGCs in *A. albensis* strain C1-4G’s genome. This identified five regions of the genome which had genes predicted to be involved in biosynthesis (Fig. 2). Two regions encoded standalone genes, a polyprenyl synthase gene which can encode a protein for terpene precursor synthesis on contig 4 and a *β*-ketoacyl synthase gene, which was predicted to be linked to aryl polyene synthesis, on contig 12. Another region had two additional *β*-ketoacyl synthase genes, also linked to aryl polyene synthesis on contig 9 (Fig. 2). Aryl polyene compounds have been linked to several functions in bacteria, including protection from oxidative stress and biofilm formation [36,37]. The results also showed a BGC which codes for the production of a siderophore on contig 16, predicted to encode baumannoferrin A or B (Fig. 2) [38]. Siderophore production is widespread, with genes encoding baumannoferrin being widespread across *Acinetobacter* spp., including *A. baumannii* [39]; which use both acinetobactin and baumannoferrin to grow under iron-limiting conditions.

AntiSMASH also identified a *ycaO* gene on contig 16, which is known to be involved in post-translational modification of multiple classes of ribosomally synthesized and post-translationally modified peptides (RiPPs) natural products [40]. This *ycaO* gene was found directly downstream of a small uncharacterized gene, representing a typical genomic context for this class of natural products. RiPPMiner [41] predicted that this upstream gene encoded the precursor peptide of a cyanobactin with an unknown structure. Cyanobactins are a diverse family of small, bioactive cyclic peptides found in cyanobacteria and some marine animals [42]. Two cyanobactins have reported antibacterial activity, Aeruginazole A with an MIC of 2.2 µM against *Bacillus subtilis* [43] and Kawaguchipeptin B with an MIC of 1 µg ml^−1^ against *Staphylococcus aureus* [44]. Finally, a BGC with no known matches was identified and predicted to encode genes for the synthesis of a *β*-lactone type compound (Fig. 2).

Our study describes the third *A. albensis* genome, showing the consistent presence of hydrocarbon degrading enzymes and a BGC encoding baumannoferrin. The genome also contains a novel arsenic resistance transposon Tn*7860* and BGCs that could encode novel beta-lactone and cyanobactin-type natural products.

## Abbreviations

ANI: average nucleotide identity
BGCs: biosynthetic gene clusters
BHI: brain heart infusion
MGE: mobile genetic elements
NCBI: National Center for Biotechnology Information
RiPPs: ribosomally synthesized and post-translationally modified peptides
